# Contemporary review on spontaneous coronary artery dissection: insights into the angiographic finding and differential diagnosis

**DOI:** 10.3389/fcvm.2023.1278453

**Published:** 2023-11-27

**Authors:** M. Kovacevic, M. Jarakovic, A. Milovancev, M. Cankovic, M. Petrovic, M. Bjelobrk, A. Ilic, I. Srdanovic, S. Tadic, D. Dabovic, B. Crnomarkovic, N. Komazec, N. Dracina, S. Apostolovic, D. Stanojevic, V. Kunadian

**Affiliations:** ^1^ Faculty of Medicine, University of Novi Sad, Serbia; ^2^ Cardiology Clinic, Institute of Cardiovascular Diseases of Vojvodina, Sremska Kamenica, Novi Sad, Serbia; ^3^ Faculty of Medicine, University of Niš, Niš, Serbia; ^4^ Clinic for Cardiology, Clinical Center Niš, Niš, Serbia; ^5^ Faculty of Medical Sciences, Translational and Clinical Research Institute, Newcastle University, Newcastle upon Tyne, United Kingdom; ^6^ Cardiothoracic Centre, Freeman Hospital, Newcastle upon Tyne Hospitals NHS Foundation Trust, Newcastle upon Tyne, United Kingdom

**Keywords:** spontaneous coronary artery dissection, pregnancy, fibromuscular dysplasia, women’s health, MINOCA

## Abstract

Spontaneous coronary artery dissection (SCAD), although in the majority of cases presents as an acute coronary syndrome (ACS), has different pathophysiology from atherosclerosis that influences specific angiography findings and enables most patients to be solved by optimal medical therapy rather than percutaneous coronary intervention (PCI). Therefore, accurate diagnosis is essential for adequate treatment of each patient as management of SCAD differs from that of ACS of atherosclerotic aetiology. So far, invasive coronary angiography remains the most important diagnostic tool in suspected SCAD. However, there are ambiguous cases that can mimic SCAD. In this review, the authors summarize current knowledge about the diagnostic algorithms, particularly angiographic features of SCAD, pitfalls of angiography, and the role of intracoronary imaging in the context of SCAD diagnosis. Finally, apart from the pathognomonic angiographic features of SCAD that are thoroughly discussed in this review, the authors focus on obscure angiography findings and findings that can mimic SCAD as well. Differential diagnosis and the timely recognition of SCAD are crucial as there are differences in the acute and long-term management of SCAD and other causes of ACS.

## Introduction

Spontaneous coronary artery dissection (SCAD) is an important cause of myocardial infarction (MI) and sudden cardiac death in young adults, particularly women. It is defined as spontaneous, acute, or subacute development of an intramural hematoma (IMH) with or without a tear of the tunica intima, leading to the formation of a false lumen that is not caused by atherosclerosis, trauma, or coronary manipulation. Compression of the true lumen leads to coronary insufficiency and typically presents with symptoms and signs of acute coronary syndrome (ACS) (1–3).

SCAD was first described in 1931 in the autopsy of a 42-year-old woman who died after a violent retching attack (4). Over the following decades, only isolated cases of SCAD were described, and with the development of invasive diagnostic and therapeutic procedures, it turned out that SCAD is much more frequent and challenging to diagnose and treat than previously thought (2).

Establishing an accurate diagnosis of SCAD as a cause of MI is challenging but, at the same time, crucial, given the different therapeutic approach compared to atherosclerotic ACS both acutely and in long-term follow-up (5, 6). Currently, invasive coronary angiography (ICA) is the gold standard for the diagnosis of SCAD, especially when combined with intracoronary imaging. However, it is associated with considerable risk of intramural hematoma and dissection propagation. Therefore, being non-invasive, computed tomographic coronary angiography (CTCA), with the improvement in techniques and protocols in recent times, has been emerging as a valid alternative to ICA for both diagnosis and even more for the follow-up (7, 8). Still, the main limitation of CTCA is the lower spatial resolution, which limits the evaluation of the distal segments of the coronary arteries, which are often affected in SCAD (9, 10). In addition, SCAD is a common coronary aetiology in the setting of MINOCA (Myocardial Infarction with Non-Obstructive Coronary Arteries) and cardiac magnetic resonance (CMR) could be useful to determine the nature of myocardial injury due to SCAD or other coronary differential diagnoses (11, 12).

Regarding the management, current European and American experts' consensus documents on SCAD recommend conservative treatment whenever possible, given the lower angiographic success and a higher complication rate of percutaneous coronary intervention (PCI) compared to those obtained in atherosclerotic disease (5, 13). Moreover, conservative treatment is associated with complete coronary healing in most cases and subsequently followed with favourable outcome (1, 5, 13–15). However, when indicated, particularly in SCAD patients presenting with STEMI and impaired coronary flow, PCI is inevitable, and effective in the substantial majority of patients, with similar in-hospital mortality and even better long-term outcomes compared with PCI for atherothrombotic STEMI (16, 17). These findings support the value of PCI in selected patients with SCAD.

In this review article, the authors summarize the current knowledge about the aetiology, epidemiology, pathophysiology, clinical presentation, risk factors, diagnostic algorithm, specifically the angiographic findings in SCAD, the angiographic pitfalls, the role of intracoronary imaging in the context of the diagnosis of SCAD and the currently recommended treatment.

### Epidemiology

According to available data, SCAD is estimated to account for 1% to 4% of ACS cases overall, up to 35% of ACS events in women younger than 50 years (16), and 23% to 68% of ACS in pregnancy (18). SCAD has been reported, although rarely, in both young adults (under 25 years) and teenagers, especially if there is no pregnancy or hereditary connective tissue disease, and it is also scarce in very old patients (over 80 years) (19). The true prevalence and incidence in the general population is, for now, unknown. With technological advances and physician awareness of SCAD as a possible cause of ACS, its existence is increasingly being recognized (20).

### Pathophysiology

The pathophysiology of SCAD is still hypothetical, and two mechanisms of its occurrence have been proposed based on imaging techniques and histopathology. The first “inside-out” mechanism explains that the tear in the tunica intima is responsible for the entry of blood and the separation of the tunica intima and tunica media. The second, more probable “outside-in” mechanism, explains that rupture of the vasa vasorum in the tunica adventitia is responsible for bleeding in the arterial wall leading to the formation of intramural hematoma (IMH). Either of these two mechanisms leads to acute or subacute false lumen formation which expands both longitudinally and circumferentially and compresses the true lumen leading to coronary ischemia and acute MI (21, 22).

### Anticipating SCAD before coronary angiography

Although the definite diagnosis of SCAD can be made exclusively by performing coronary angiography, with or without the aid of intravascular imaging, there are some inciting factors, associated conditions, and precipitants that will point to possible SCAD diagnosis before coronary angiography is done. In particular, the link between female gender, pregnancy, fibromuscular dysplasia (FMD) and SCAD has been established in multiple series.

#### Female gender

According to available data collected from observational studies (Table 1), more than 90% of patients with SCAD are perimenopausal women, with an average age of 47–53 years, and a high percentage (90%) of associated FMD. Occurrence in men is less studied and shows different risk factors than in women, with 44% of cases associated with heavy lifting or isometric exercise. Men also report fewer traditional female-associated risk factors for SCAD, such as depression, anxiety, emotional stress, and migraines (34, 35).

**Table 1:** The main contemporary case series with spontaneous coronary artery dissection (SCAD), clinical and angiographic presentation, initial management and the outcome.

Authors	Year	Patients *N*	Female gender *N* (%)	Initial presentation STEMI/ NSTEMI *N* (%)	SCAD type 1/2/3/4 *N* (%)	ICI total N (%)	Initial medical therapy *N* (%)	Crossover to revasc. *N* (%)	PCI CABG *N* (%)	In-hospital mortality *N* (%)	FU	FU mortality *N* (%)	Recurrent SCAD *N* (%)
Alfonso et al. (15)	2012	45	9 (82)	18 (40) 16 (36)	NA	14 (31.1)	36 (80)	7 (19.4)	8 (17.8) 1 (2.2)	1 (2.2)	730 day^a^	1 (2.2)	2 (4.4)
Saw et al. (23)	2014	168	155 (92.3)	44 (26.1) 124 (73.9)	59 (29.1) 136 (67) 8 (3.9)	NA	139 (82.7)	6 (4.3)	28 (16.7) 1 (0.59)	0	6.9 years^a^	4 (2.4)	22 (13.1)
Tweet et al. (14)	2014	189	174 (92)	111 (58.7) 77 (40.7)	NA	24 (13)	94 (49.7)	8 (8.5)	89 (47.1) 6 (3.2)	1 (0.53)	2.3 years^a^	1 (0.5)	27 (14.3)
Lettieri et al. (24)	2015	134	109 (81)	66 (49.2) 54 (40.3)	NA	NA	78 (58.2)	2 (2.6)	51 (38.1) 5 (3.73)	3 (2.2)	22 months^a^	8 (5.9)	6 (4.7)
Nakashima et al. (16)	2016	63	59 (94)	55 (87) 8 (13)	27 (43) 35 (55) 1 (2)	41 (65.1)	28 (44.4)	0	34 (54) 1 (1.6)	NA	50 month^a^	1 (0.63)	17 (27)
McGrath-Cadell et al. (25)	2016	40	38 (95)	12 (30) 26 (65)	NA	NA	27 (67.5)	0	12 (30) 1 (2.5)	0	16 month	0	3 (8)
Faden et al. (26)^b^	2016	79	79 (100)	42 (53.2) 24 (30.4)	NA	NA	27 (34.2)	NA	31 (39.2) 23 (29.1)	3 (3.8)	NA	NA	NA
Rogowski et al. (27)	2017	64	60 (94)	19 (30) 44 (69)	32 (47.8) 32 (52.2)	NA	56 (87.5)	0	7 (10.9) 1 (1.6)	1 (1.6)	5.9 years^a^	0	3 (4.7)
Saw et al. (28)	2017	327	297 (90.8)	84 (25.7) 243 (74.3)	99 (25.6) 270 (69.8) 18 (4.7)	NA	272 (83.2)	9 (3.3)	54 (16.5) 7 (2.14)	0	3.1 years^a^	1 (0.3)	9 (2.8)
Clare et al. (29)	2019	208	185 (88.9)	41 (19.7) 167 (80.3)	NA	NA	176 (84.6)	NA	9 (4.3) 23 (11.1)	NA	1 years	5 (2.4)	22 (10.6)
Mori et al. (30)	2021	302	267 (88.4)	145 (48) 136 (45)	52 (17.2) 149 (49.3) 20 (6.6) 81 (26.8)	91 (30.1)	198 (65.6)	NA	100 (33.1) 4 (1.3)	1 (0.3)	22 month^a^	1 (0.3)	10 (3.3)
Saw et al. (31)	2022	750	664 (88.5)	223 (29.7) 524 (69.9)	291 (29) 603 (60.2) 108 (10.8)	57 (7.6)	648 (86.4)	15 (2.3)	104 (13.9) 5 (0.7)	1 (0.1)	3 years	5 (0.7)	18 (2.4)
Garcia Guimaraes et al. (32)	2022	389	344 (88)	211 (54) 156 (40)	84 (19) 271 (62) 38 (9) 48 (11)	93 (24)	305 (78.4)	10 (3.3)	84 (21.6) 0 (0%)	7 (1.8)	29 month	9 (2.5)	7 (2)
Jensen et al. (33)	2023	186	108 (81)	80 (43) 106 (57)	NA	NA	134 (72)	NA	43 (23.1) 9 (4.8)	NA	4.5 years^a^	9 (4.8)	42 (22.6)

The Table reports all studies that included more than 40 patients.

STEMI, ST segment elevation myocardial infarction; NSTEMI, Non ST segment elevation myocardial infarction; SCAD, spontaneous coronary artery dissection; PCI/CABG, percutaneous coronary artery intervention/coronary artery bypass graft; MI, myocardial infarction; FU, follow-up; NA, not applicable; y, year, m, month; d, day;.

^a^ median.

^b^ SCAD in pregnancy; ICI, intracoronary imaging.

#### Pregnancy and sex hormones

Pregnancy-related SCAD accounts for approximately 10% of SCAD cases. However, one-third of ACS in pregnancy and almost half of ACS in the postpartum period are due to SCAD. Furthermore, most pregnancy-related SCAD occur in the first week after delivery, when estrogen and progesterone levels decline (3, 36). This association with pregnancy highly suggest a pathophysiological role of female sex hormones. However, this hormonal hypothesis has been challenged by a few studies demonstrating that the rate of hormonal contraception, hormone replacement therapy, nulliparity and multiparity did not differ between SCAD patients and the general population (13, 37). It is also unclear whether the absolute levels or fluctuations in circulating estrogen and progesterone influence the SCAD the most. Furthermore, estrogen level reduction in the premenstrual, late luteal phase, has been studied in patients with coronary vasospasm and migraines (38, 39). The precise nature of this relationship remains to be elucidated but may relate to changes in the intima-media composition, vessel microvasculature or vascular connective tissue.

#### Systemic connective tissue diseases

Patients with systemic connective tissue diseases associated with arteriopathy or arterial dissection, such as Marfan, Ehlers-Danlos, and Loeys-Dietz syndrome, account for less than 5% of SCAD patients (6). According to registries, more than 50% of patients with SCAD who underwent imaging for extra-coronary vascular abnormalities have FMD. It is defined as a non-inflammatory, non-atherosclerotic condition diagnosed primarily in women and characterized by abnormal proliferation of one or more layers of the arterial wall, resulting in arterial stenosis, dissection, and aneurysms of medium-sized arteries. Other vascular findings in patients with SCAD include cerebral and visceral aneurysms, dissections, pseudoaneurysms, and arterial tortuosity in patients with and without diagnostic criteria for FMD. Analysis of several cohort studies concluded that systemic inflammatory diseases are associated with SCAD in less than 5% of cases, unlike FMD (19, 40).

#### Genetics

Although genetic predisposition is suggested in a very small number of cases, including first- and second-degree relatives, SCAD does not appear to be a strongly inherited condition. The association of SCAD with congenital connective tissue diseases and arteriopathies has been described, however, genetic mutations are rare and are most often expressed in Ehlers-Danlos, Loeys-Dietz, Marfan syndrome, Autosomal dominant polycystic kidney disease and Pseudoxanthoma elasticum. Although no single SCAD gene has been described yet, research has identified individual risk loci with potential genes that carry a biological and pathophysiological risk, including those associated with FMD and other vascular disorders. Routine genetic testing is not currently recommended but may be considered in SCAD survivors with suspected connective tissue diseases or hereditary arteriopathies (3, 41).

#### Migraines

The results of several studies have shown that endothelial dysfunction in migraine plays a role in conditions such as stroke and cervical arterial dissection, which correlates with the pathophysiology of SCAD (42, 43).

#### Emotional or physical stress as precipitating factors

Up to two-thirds of patients with SCAD have a history of stressors that preceded chest pain. In women, it is most often emotional stress, while in men it is most often physical stress, including isometric exercises and heavy lifting. One hypothesis is that these precipitating factors lead to a catecholamine storm, which increases coronary afterload leading to intimal rupture or vasa vasorum disruption (44, 45). Similar pathophysiologic mechanism is believed to be responsible for Takotsubo cardiomyopathy, influencing some overlap in the clinical presentations of these two entities. Moreover, there are described cases with both conditions in the same setting (45, 46).

### Clinical presentation

SCAD most commonly presents with chest pain and other common symptoms of ACS, with electrocardiographic changes directing to MI with ST-segment elevation (STEMI) registered in 26-58.7% of cases overall and up to 75% in pregnancy-associated SCAD (14, 28, 47, 48). However, SCAD can also present as cardiogenic shock, ventricular arrhythmias or sudden cardiac death (49, 50). SCAD patients are younger, more often female, and have fewer traditional cardiovascular risk factors than patients with atherosclerotic ACS. Pregnancy-related SCAD has a more severe clinical course and usually presents as STEMI, particularly anterior, with left main and multivessel involvement (48, 51). Therefore, resulting in a more extensive myocardial injury, it is associated with an increased incidence of cardiogenic shock requiring mechanical circulatory support, and cardiac transplantation, leading to a higher maternal and fetal mortality rate (48). The presence or absence of traditional cardiovascular risk factors is not very useful for examining the likelihood of SCAD. Despite the low burden of common risk factors compared to atherosclerotic ACS, patients with SCAD are not free of them. The prevalence of hypertension is about 30%, dyslipidemia is present in a range of 20%–35%, while diabetes is uncommon (less than 5%) (37, 48, 52). However, it is documented that the younger the patient is and the lower the number of traditional risk factors, the greater the probability of SCAD (52).

### Angiographic finding in SCAD

To establish the diagnosis of SCAD, apart from common ACS clinical presentation and predisposing factors such as female gender and FMD that can increase the likelihood of SCAD, coronary angiography with or without adjunctive intravascular imaging is still crucial for accurate diagnosis. Nevertheless, three typical angiographic patterns of SCAD were proposed by Saw to aid the diagnosis (22, 53).

**Figure 1:**
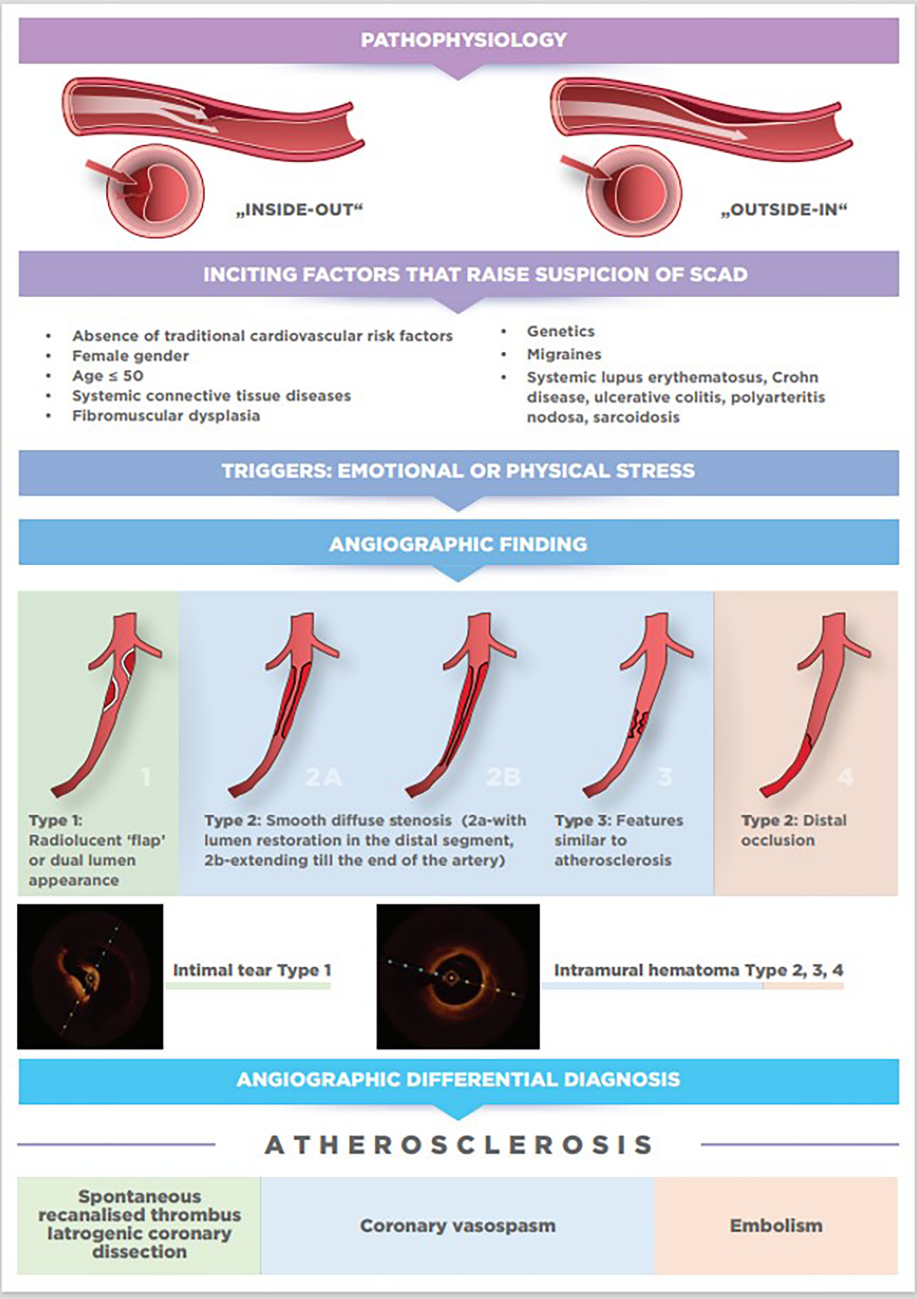
From pathophysiology to diagnosis.

**Figure 2:**
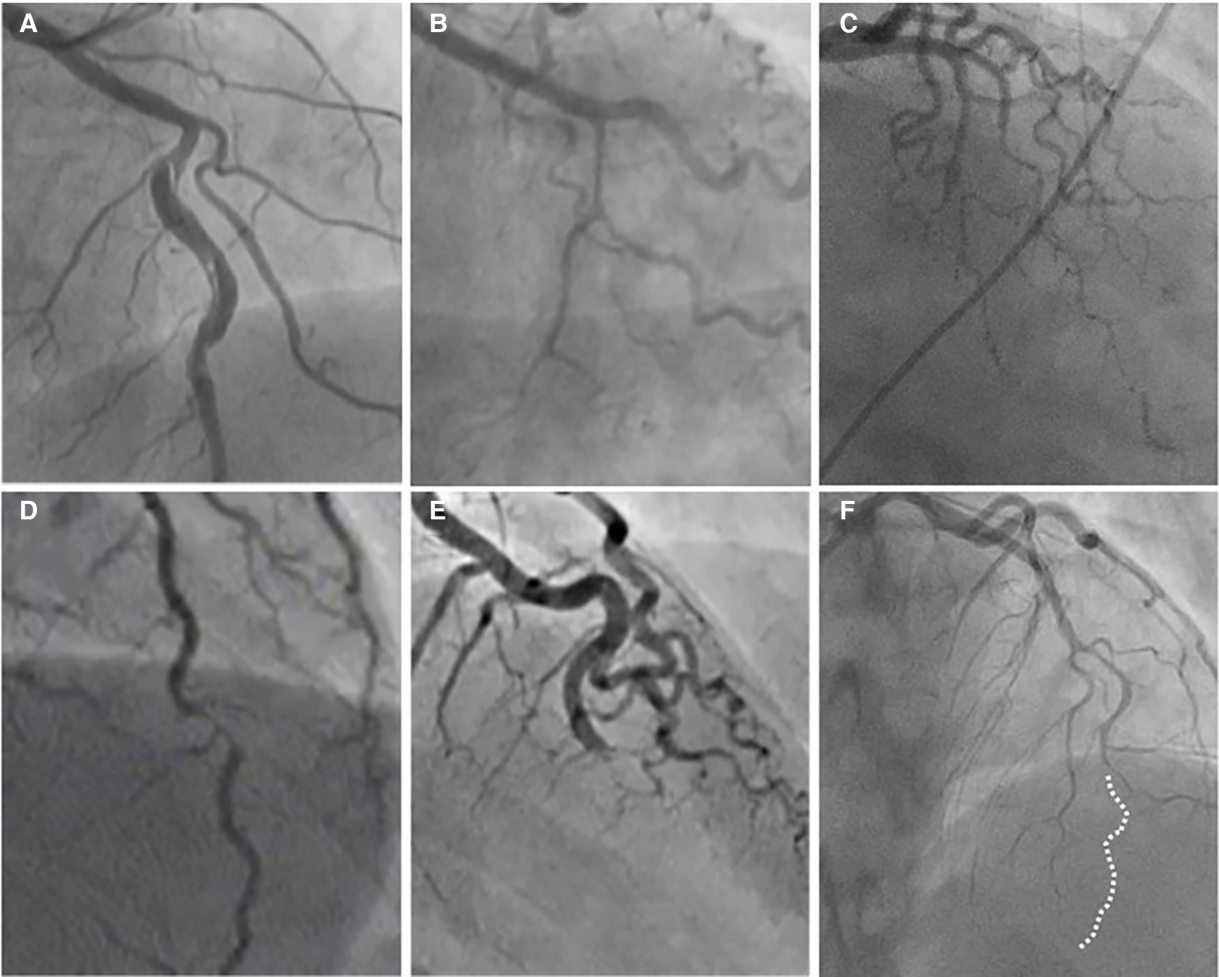
The angiographic appearance of SCAD. (**A**) Type 1-recognizable radiolucent flap; (**B**) type 2a-smooth diffuse stenosis with lumen restoration in the distal segment; (**C**) type 2b-smooth diffuse stenosis extending till the end of the artery; (**D**) type 3-resembling atherosclerosis; (**E**) type 4-distal occlusion; (**F**) an example of hybrid type SCAD- Type 2 in mid to distal segment with the transition to the Type 4-distal occlusion (dotted line depicts missing LAD).

*Type 1* accounts for about one-third of cases (16, 54), and represents the pathognomonic finding with multiple radiolucent lumen of linear filling defect (recognizable true and false lumen), usually with contrast dye staining in the false lumen. This appearance of SCAD is caused by the presence of an intimal tear which is identified in approximately 30% of SCAD cases (Figure 1, Figure 2A).

*Type 2* is the most common pattern (two-thirds of cases, Table 1) (16, 54), characterized by an abrupt change in the arterial calibre causing long and smooth stenosis caused by IMH, that tapers distally. It is located predominantly in the transition from mid to distal segments, most frequently affecting LAD. It is divided into Type 2a when there is restoration of the normal vessel in the distal segment (Figure 1, Figure 2B), and Type 2b, when the stenosis extends till the end of the artery (Figure 1, Figure 2C).

*Type 3* is the least common (less than 5%) (16, 54), resembles atherosclerotic plaque with underlying focal, more localized IMH, thus difficult to diagnose without the assistance of intravascular imaging (Figure 1, Figure 2D).

This Yip-Saw classification (22) is mainly focused on the most common angiographic findings and is particularly helpful in recognizing Type 2 SCAD once interventional cardiologists become familiar with the pattern. Some authors, however, prefer the pathological description (presence of intimal tear or *fenestrated SCAD* vs. IMH or *non-fenestrated SCAD*) over Yip-Saw “type” classification, given the finding of a retrospective studies showing that isolated IMH (corresponding to angiographic SCAD type 2 and 3) carries a higher risk of SCAD extension and clinical deterioration, while intimal tear (fenestrated SCAD, angiographic type 1) may have a protective role in some patients possibly via decompression of IMH into the lumen (23, 30, 32). Although the registries have found increased incidence of MACE in patients with IMH type of SCAD the burden of evidence does not allow to discriminate this type as the one with higher risk of events. Detailed evaluation with intracoronary imaging is needed to define its type and to identify high-risk features associated with more adverse events. Furthermore, SCAD is a highly dynamic process, fenestrated and non-fenestrated SCAD may be considered as two distinct pathological manifestations of the same substrate, with IMH that may precede intimal tear, which is consistent with the “outside-in” theory of SCAD occurrence. Therefore, for better understanding and decision-making process, Yip-Saw classification (22) is the preferred one.

Recently, additional Type 4 SCAD has been proposed to describe total occlusion, usually of a distal vessel, a pattern particularly challenging to diagnose (Figure 1, Figure 2E) (55).

However, all these types can coexist in the same vessel, generating hybrid types (Figure 2F).

Although SCAD has been reported in all coronary arteries, sporadically even simultaneously (contiguous or non-contiguous), LAD is the most affected artery (5, 24, 54). Regarding coronary segments, SCAD has a predilection for more distal coronary segments (5, 54) in contrast to atherosclerosis, particularly Type 2 and 4 SCAD. On the contrary, type 1 SCAD generally affects proximal segments. Another angiographic feature favouring SCAD is the absence of atherosclerotic lesions in coronaries unaffected by SCAD (5, 52). Furthermore, the angiographic ambiguity of SCAD is constrained by side branches, which appear to provide resistance to further longitudinal extension (52). It is also demonstrated that SCAD happens more often in patients with tortuous arteries. Moreover, severe tortuosity (≥2 consecutive curvatures ≥180°) was associated with a three times higher risk of recurrent SCAD (10).

### Differential diagnosis of SCAD

Although the angiographic features of SCAD are characteristic, several potential pitfalls and essential differential diagnoses should be considered.

**Figure 3:**
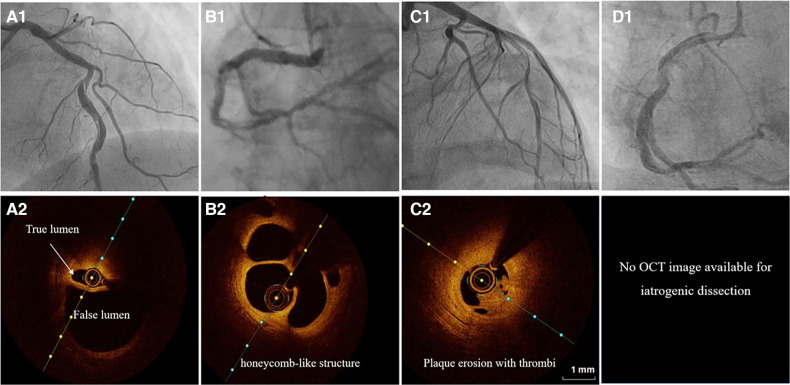
The angiographic differential diagnosis for type 1 SCAD. (**A1**) Type 1 SCAD with linear filling defect in mid-LAD. (**A2**) OCT finding in Type 1 SCAD- clear evidence of a small true (arrow) and a big false lumen with the OCT probe situated in the true lumen. (**B1**) Angiographic finding in spontaneous recanalized coronary thrombus (SRCT) in the mid and distal right coronary artery resembles SCAD. (**B2**) OCT finding in SRCT depicts a typical honeycomb-like structure. (**C1**) Angiographic finding in atherosclerotic acute coronary syndrome with plaque erosion and subsequent thrombus apposition. (**C2**) OCT finding corresponding to panel C1 with evidence of plaque erosion and intraluminal thrombi. (**D**) Angiographic finding in iatrogenic coronary dissection caused by guiding catheter, a picture resembling SCAD Type 1.

Type 1 angiographic appearance of SCAD is pathognomonic, usually developing in the late disease course, probably due to decompression of the false lumen hematoma into the true lumen. However, this angiographic finding has several mimickers, such as spontaneous recanalized coronary thrombus (SRCT) (56, 57) (Figures 3, panels B1, 2), atherosclerotic plaque rupture or erosion with apposition of thrombi (Figures 3, panels C1, 2), or even iatrogenic coronary dissection (Figures 3, panel D).

SRCT is a rare condition characterized by multiple communicating channels divided by thin septa, usually termed a “honeycomb-like” structure, “lotus root” appearance, or “Swiss cheese” pattern. The proposed mechanism of SRCT is the recanalization of an in-situ thrombus, formating several lumens which differ in size. To distinguish these two diagnoses, high-resolution intracoronary imaging techniques, intravascular ultrasound (IVUS) or optical coherence tomography (OCT), can be helpful (Figures 3, panels B1–2). Interestingly, “lotus root” pattern was recently observed in a patient with SCAD, possibly as a result of uncommon remodelling and healing pattern of subacute or chronic SCAD. (58)

Rupture or erosion of atherosclerotic plaque resulting in intraluminal thrombus formation can mimic type 1 SCAD as well (Figures 3, panels C1–2). Furthermore, contrast penetration into the atherosclerotic plaque core causing a localized plaque-associated dissection can resemble contrast penetration into the false lumen of a Type 1 SCAD. Although intraluminal thrombus might be seen in the occlusive (Type 4) SCAD, the presence of substantial thrombus and distal embolization should divert diagnosis to ACS caused by typical mechanisms, atherosclerotic plaque rupture or erosion. These two entities, although resembling angiographically, can be easily separated by intravascular imaging techniques (Figures 3 A1–2, C1–2).

Another feature similar in angiographic appearance to type 1 SCAD is iatrogenic coronary artery dissection (Figures 3, panel D). Furthermore, SCAD is associated with an increased risk for iatrogenic dissection (59), either due to the vulnerability of such coronary artery with predisposing arteriopathies, particularly FMD or due to the injury of thin intima with preexisting hematoma. Both deep guiding catheter intubation and the jet of contrast injection can make a tear into the vessel wall creating a typical picture of a dual (true and false) lumen. Other mimickers of SCAD type 1 include different contrast flow patterns simulating a linear filling defect, usually due to insufficient contrast volume or flow, and can easily be distinguished from SCAD by an experienced interventional cardiologist and by giving a more fulsome, generous contrast injection.

**Figure 4:**
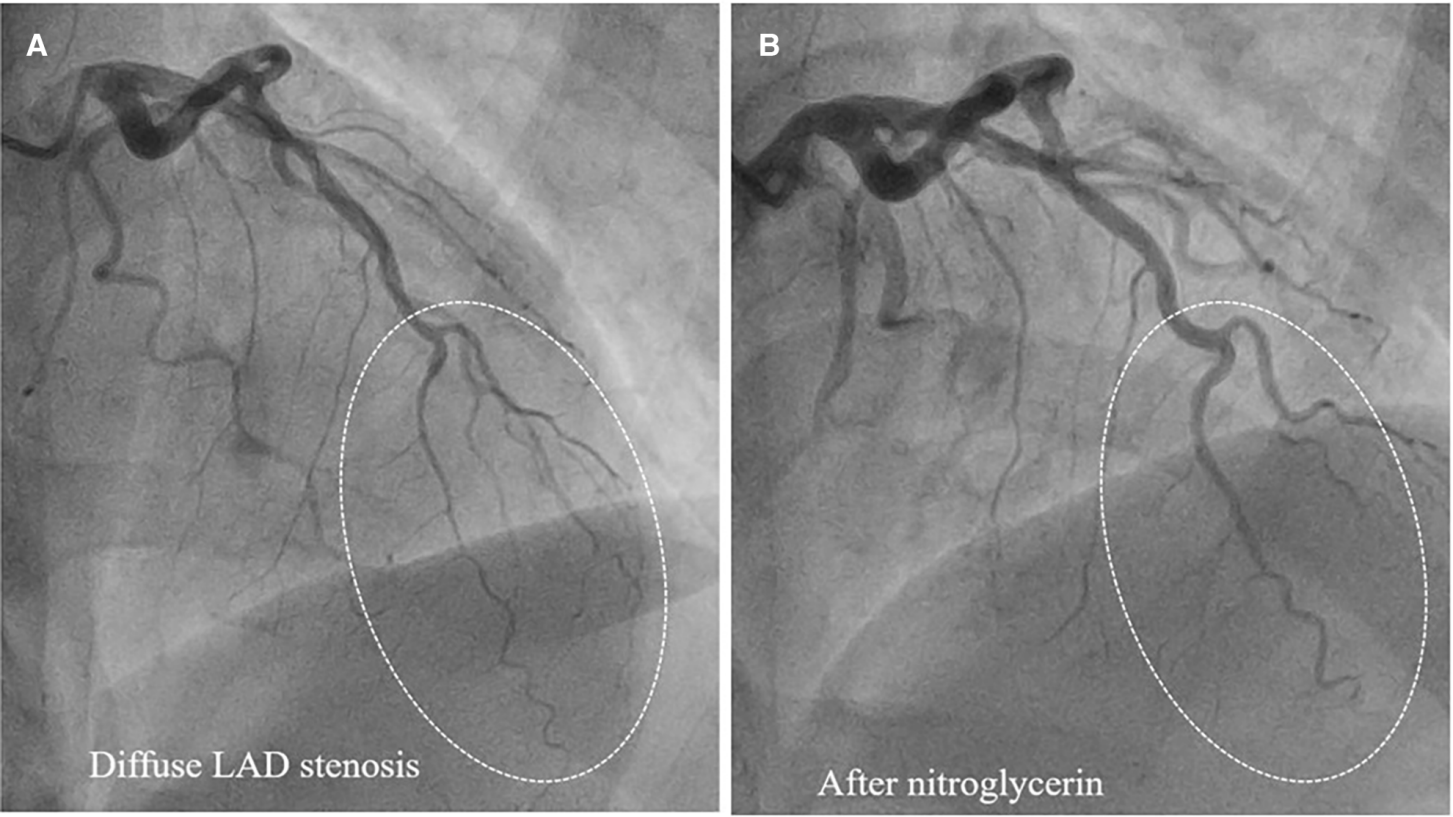
Epicardial coronary vasospasm mimics type 2 SCAD. **(A**) Diffuse LAD stenosis resembling Type 2 SCAD. (**B**) Restoration of vessel lumen after nitroglycerin administration confirms the epicardial vasospasm diagnosis.

Type 2 SCAD is the most common (Table 1), angiographically displayed with long and smooth stenosis. The most common mimickers of SCAD type 2 are coronary vasospasm and atherosclerosis. Coronary vasospasm can be focal, resembling SCAD type 2a or diffuse, extending distally as in type 2b SCAD. However, intracoronary nitroglycerine administration can reveal coronary vasospasm without difficulties (Figure 4).

**Figure 5:**
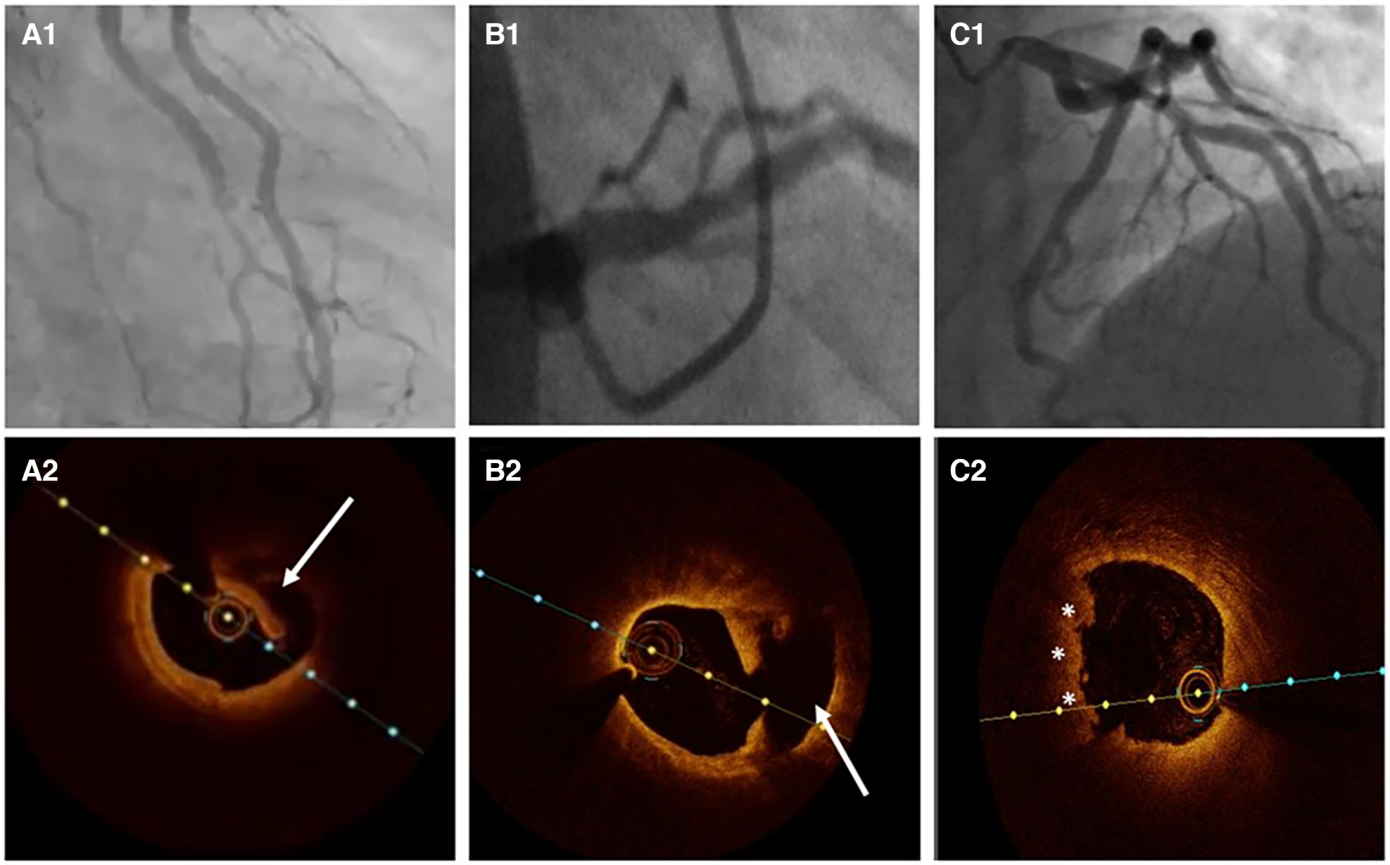
Atherosclerosis versus SCAD. (**A1**) Ramus intermedius lesion resembling atherosclerotic plaque rupture. (**A2**) OCT image demonstrating SCAD with intima-media complex dehiscence (arrow). (**B1**) Left anterior descending (LAD) lesion in proximal segment. (**B2**) OCT evidence of atherosclerotic plaque rupture (arrow). (**C1**) Haziness in proximal LAD. (**C2**) Plaque erosion with apposition of thrombi (asterisks).

**Figure 6:**
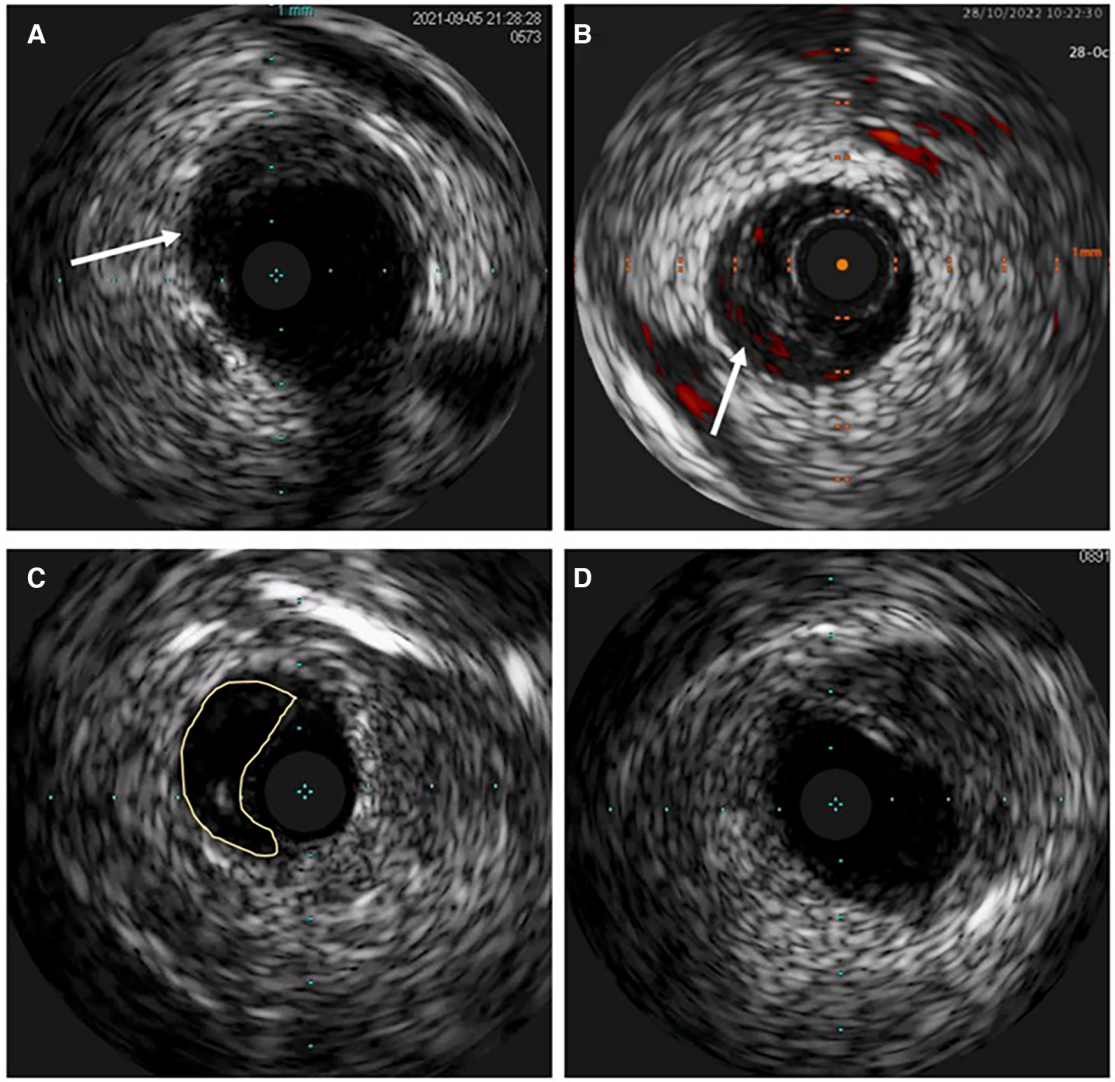
Intravascular ultrasound (IVUS) image of spontaneous coronary artery dissection (SCAD). (**A**) Intramural hematoma with the dissection entry (arrow). (**B**) ChromaFlow highlighting blood flow within intramural hematoma (IMH) with red color (arrow). (**C**) True and false lumen (yellow field) with the IVUS probe in the true lumen. (**D**) IMH in the left main resembles lipid-rich atheroma. Careful analysis of the entire pull-back length may be required in such cases.

**Figure 7:**
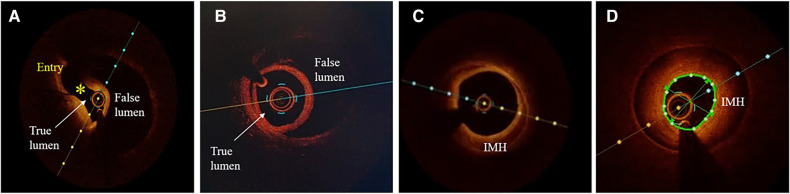
Optical coherence tomographic (OCT) imaging of SCAD. (**A**) A typical picture of the true and false lumen with visible dissection entry (asterisk). (**B**) True and false lumen without connection and with OCT probe situated within the true lumen. (**C**) IMH with incomplete dehiscence of the true lumen from the vessel wall. (**D**) Circumferential intramural hematoma (IMH) with complete dehiscence.

Atherosclerosis is the most common differential diagnosis of Type 2, particularly Type 3 SCAD. Short stenosis with underlying hematoma in Type 3 SCAD is often misdiagnosed by coronary angiography unless an intravascular imaging technique is used (Figure 5). Intravascular ultrasound (IVUS) and optical coherence tomography (OCT), each with specific advantages and disadvantages, are valuable for diagnostic uncertainties. IVUS, as the first intravascular imaging device that was introduced in 1980s (60), has greater depth penetration, enabling complete visualization of the vessel wall to the external elastic lamina. At the same time, it has limited spatial resolution (150 μm) and is insufficient to distinguish SCAD from lipid-rich atheroma and for identification of subtle features associated with SCAD (intimal-medial membrane, small fenestrations between true and false lumens) (Figure 6). A typical IVUS feature, the white-black-white appearance (1) of the intimal-medial membrane, is pathognomonic for SCAD but not often seen. However, the main advantage of IVUS is that complete blood clearance with high-pressure contrast injection is not required. On the other side, OCT has the edge over IVUS due to the higher spatial resolution (15 μm), which enables to identify SCAD related features (61), distinguishing true and false lumen, the extent of the false lumen, whether it is circumferential or not, the “entry points” connecting true and false lumen, presence of intraluminal thrombi (Figure 7). The main pitfall of OCT is the necessity of blood clearance with a high-pressure contrast injection which portends the risk of false lumen extension, particularly in Type 1 SCAD.

**Figure 8:**
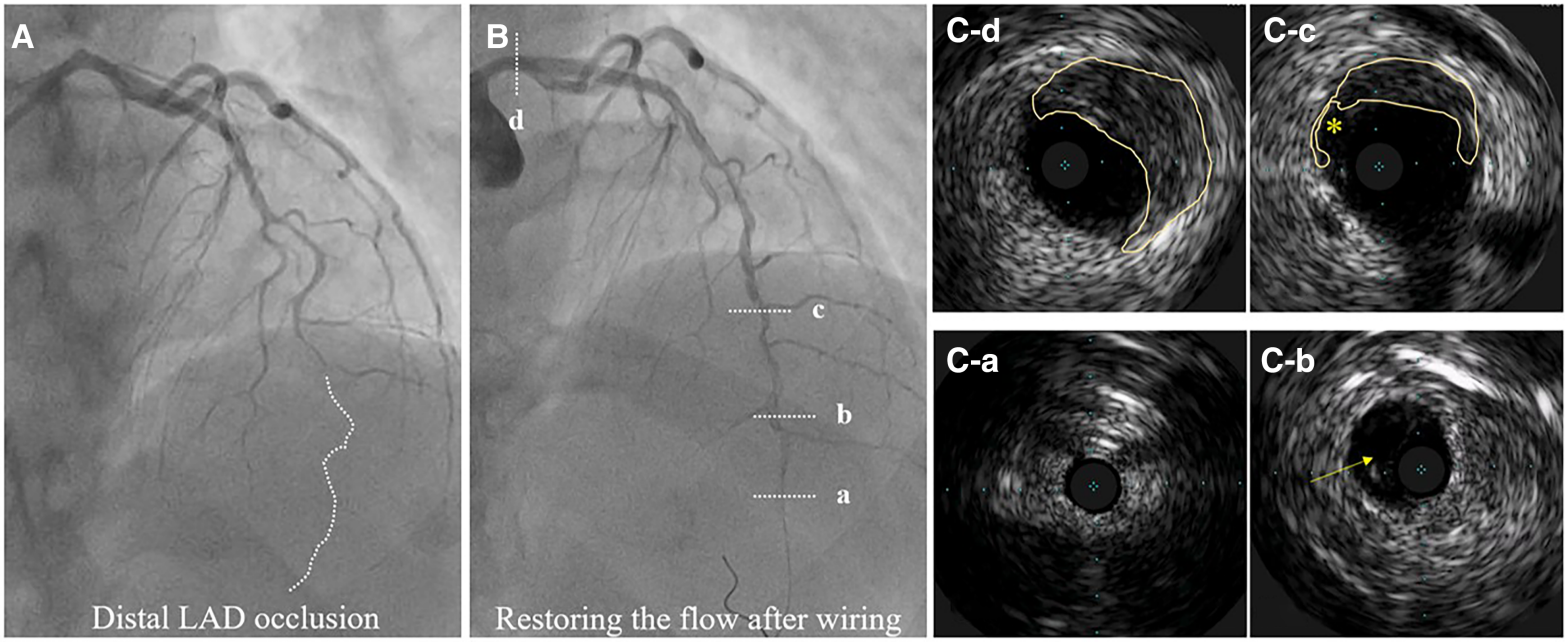
Hybrid SCAD diagnosed by IVUS. (**A**) Baseline angiography, dotted line depicts missing distal left anterior descending (LAD) artery. (**B**) flow restauration after wiring. (**C-a**) Distal, not diseased LAD. (**C-b**) Distal LAD with a visible true and false lumen. (**C-c**) IMH from 9 to 3 o’clock and dissection entry (*). (**C-d**) IMH in the left main (from 10 to 5 o’clock).

**Figure 9:**
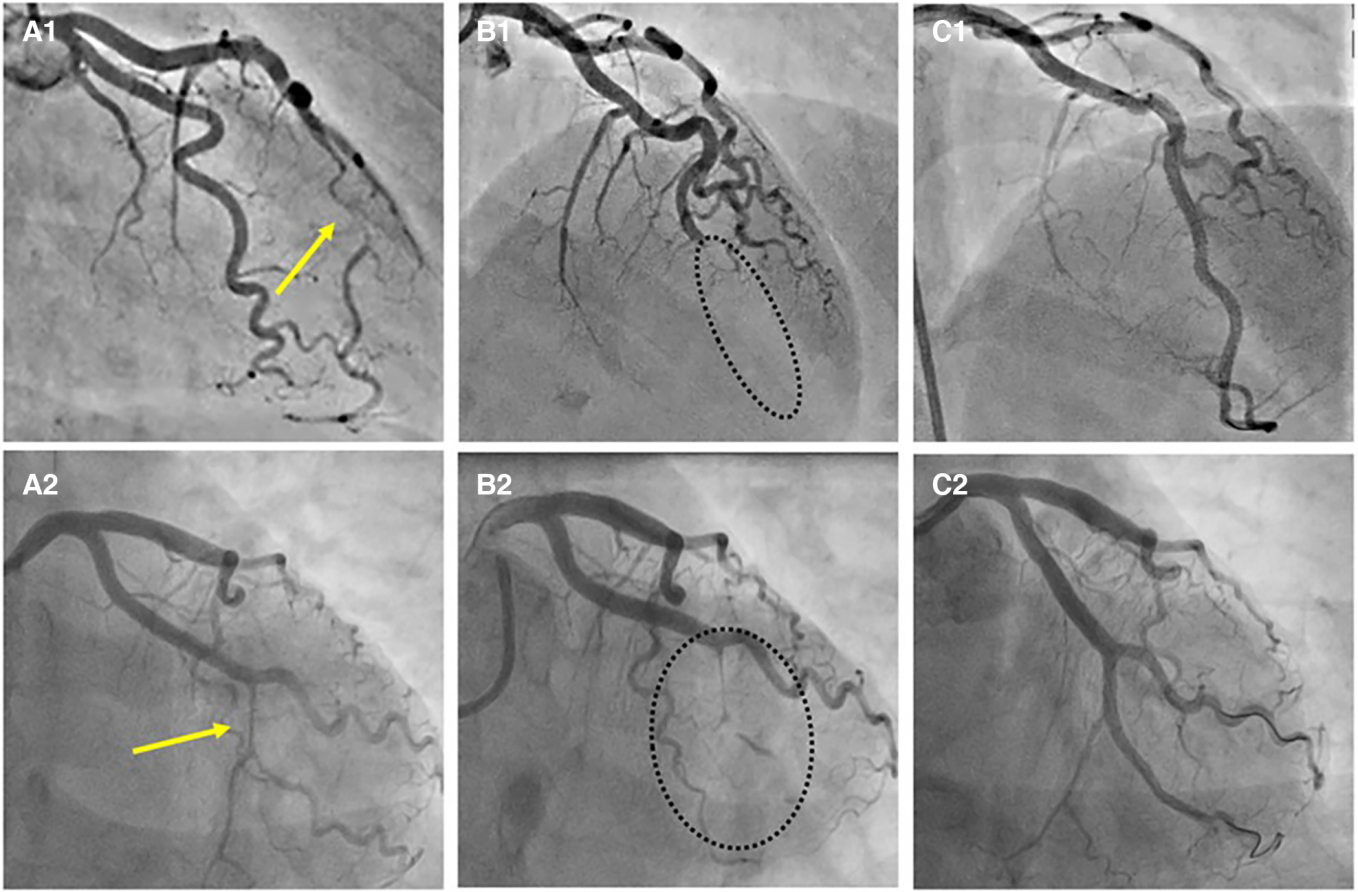
Progression of SCAD. (**A1**) SCAD Type 2a in distal left anterior descending (LAD) artery (arrow), referred for OMT. (**A2**) SCAD Type 2a in left circumflex (LCX) artery (arrow), referred for OMT. (**B1,B2**) Progression to SCAD Type 4 during watchful waiting strategy-dotted lines depict missing LAD (B1) and missing LCX (B2). (**C1, C2**) Final result after PCI.

Recently described, Type 4 SCAD, characterized by a total occlusion of a distal vessel, is particularly ambiguous, usually misdiagnosed as atherosclerotic plaque rupture with thrombus formation as in STEMI and thus systematically treated with PCI. Coronary embolization from an upstream source of thrombi, such as prosthetic, mechanical valves or rheumatic valves, coronary aneurysms, or paradoxical embolization, can mimic Type 4 SCAD as well. Nevertheless, thorough anamnesis, inciting risk, and precipitating factors can raise suspicion of SCAD. Restoration of blood flow after wiring the artery can unmask typical SCAD features and, if combined with intracoronary imaging techniques, might enable definite SCAD diagnosis. If treated conservatively afterwards, complete vessel healing follows the natural SCAD process. Additionally, Type 4 frequently coexists with other types, either following Type 1, which can be the source of an embolus or continuing to other types, in which case IMH proximal to the occlusion can be detected by intravascular imaging techniques (Figure 8). SCAD progression from Type 1, 2 or 3 to Type 4 is also possible, particularly during a watchful waiting strategy in severe forms of SCAD (Figure 9).

### Management

Given the complex pathophysiology of SCAD and the natural tendency for spontaneous healing, conservative management is the recommended strategy in stable SCAD. In contemporary cohorts (Table 1), conservative management was successful in more than 80% of medically managed patients (37), with angiographic evidence of healing within weeks to months (14, 27). Furthermore, revascularization with PCI is associated with a higher complication rate and a lower procedural success rate and does not protect against repeat revascularization or recurrent SCAD (14).

The goal of medical therapy early after SCAD diagnosis is to relieve the symptoms (particularly chest pain), manage blood pressure and to prevent SCAD extension and recurrence. Due to the lack of randomized trials, management is primarily based on expert consensus.

Although SCAD presents with ACS, if not treated with PCI, due to the distinct pathophysiology from atherosclerotic ACS, the use and duration of dual antiplatelet therapy (DAPT) is controversial. On the one hand, it is believed that the presence of intimal tear can be prothrombotic, influencing, though very rarely, luminal thrombus formation (62), justifying DAPT (acetylsalicylic acid and clopidogrel) in the acute phase. On the other hand, IMH propagation can be stimulated with antiplatelet and anticoagulant medication. Therefore, the general consensus is to avoid anticoagulant therapy and to shorten DAPT duration (up to 4 weeks) (6, 13) unless there is an unequivocal indication for anticoagulant treatment (atrial fibrillation, left ventricular thrombus). Long-term acetylsalicylic acid may be reasonable in patients with FMD or evidence of atherosclerosis on intravascular imaging.

Angiotensin-converting enzyme inhibitors, angiotensin receptor antagonists, mineralocorticoid receptor antagonists, and beta-blockers are recommended in SCAD patients with significant impairment of left ventricular systolic function according to heart failure guidelines (63). Due to the possible protective role of beta-blockers for SCAD recurrence, beta-blocker should be considered in all patients (3). The rationale for statin therapy in SCAD patients is unknown, and it is reserved for patients with preexisting dyslipidemia.

In hemodynamically unstable patients with ongoing ischaemia and impaired distal coronary flow, and when high-risk anatomic features (left-main involvement or multivessel SCAD) are anticipated, revascularization should be an option (5, 21). Additional risks might be encountered during PCI, from wiring the false lumen, inadequate stent sizing and expansion, iatrogenic dissection, hematoma propagation, side branch occlusion, and late stent malapposition after IMH resorption. Thus, intravascular imaging is highly endorsed to guide the procedure.

The main objective of PCI should be the restoration of blood flow mainly with plain old balloon angioplasty (POBA), preferably by a cutting balloon for hematoma fenestration and depressurizing the false lumen. If the decision to implant stent is undertaken, to avoid hematoma expansion, it is advisable to perform direct stenting, either with one longer stent or with a three-stent technique, covering distal and proximal dissections edges before stenting the intermediate segment (64). The use of bioresorbable stents may be beneficial by providing a temporary scaffolding of the vessel and avoiding late stent malapposition after IMH resorption (65).

Regarding revascularization options, PCI is recommended over coronary artery bypass grafting (CABG). The latter is reserved for PCI failure or when there is a substantial myocardium at risk (left main bifurcation involvement or multivessel SCAD) (21). In these circumstances, venous grafts are preferable, given the risk of graft failure due to the healing of the native coronary arteries and subsequent competitive flow.

Cardiogenic shock (CS) can complicate SCAD. The true prevalence of CS in SCAD patients is unknown (5, 13). However, Lobo et al. (17) reported that the prevalence of CS in SCAD presenting with STEMI is twice that of atherothrombotic STEMI (19% vs. 9%) and most often associated with left main dissection. An even higher prevalence of CS in SCAD is described in a systematic review of 120 pregnant women, with 24% presenting with cardiogenic shock and requiring mechanical circulatory support (MCS) and subsequent revascularization or heart transplantation (48). The utility of MCS in patients with SCAD is mainly based on several case reports documenting successful use of intra-aortic balloon pump (IABP), Impella, venoarterial extracorporeal membrane oxygenation (VA-ECMO), or left ventricular assist device (LVAD), either as a bridge to recovery or heart transplantation (48, 50, 66, 67).

### Outcomes and follow-up

In SCAD survivors, long-term mortality is very low (Table 1), with a 10-year survival rate of 92% in the USA Mayo Clinic series (68) to 100% survival rate in Swiss series with a median follow-up of 4.5 years (27). However, the overall major adverse cardiac events (MACE) in these patients are common but with considerable variation between published series, ranging from 14.6% of 6-year MACE in the Italian series (24) to 47.4% of 10-year MACE in the US series (68). MACE is primarily driven by target vessel revascularization in PCI-treated SCAD and SCAD recurrence. The recurrence rate has been estimated to diverge (Table 1) from 2% in a 2-year follow-up (32) to 27% in a 5-year follow-up (21). Recurrent SCAD often involves new territory and may manifest as a different angiographic type than previously. The main contributors to SCAD recurrence are hypertension (28), and severe coronary tortuosity (10), while beta-blocker use may be protective (28).

Given the known risk for catheter-induced iatrogenic dissection in SCAD patients (59), routine angiographic follow-up is not recommended. For that purpose, CCTA, although with limited potential in the diagnostic algorithm (69), can be a valuable option to confirm SCAD healing, particularly in SCAD type 1 (7, 8). However, further data is needed before CCTA can be recommended for SCAD follow-up.

## Conclusion

Spontaneous coronary artery dissection is a common cause of myocardial infarction in young adults, particularly women. Distinct from atherosclerotic ACS by pathophysiology, with several non-traditional risk factors and associated conditions that can increase the likelihood of SCAD, the final diagnosis is made by coronary angiography with or without intravascular imaging techniques. However, apart from well-known SCAD angiographic patterns, occasionally, it is challenging to distinguish it from atherosclerotic plaque rupture or erosion, coronary vasospasm, spontaneous recanalized thrombus, embolism or iatrogenic dissection. Therefore, intravascular imaging is advisable to confirm SCAD-specific features such as intramural hematoma or intimal tear with a clear recognition of true and false lumen. Finally, timely and accurate diagnosis is essential as there are differences in the acute and long-term management of SCAD and other causes of ACS, with the recommendation for conservative management of SCAD whenever possible.
